# Pulmonary Tuberculosis with Concomitant Aspergillus Fungal Ball in a Diabetic Indian Male: A Rare Case Report

**DOI:** 10.7759/cureus.41443

**Published:** 2023-07-06

**Authors:** Sankalp Yadav

**Affiliations:** 1 Medicine, Shri Madan Lal Khurana Chest Clinic, Moti Nagar, New Delhi, IND

**Keywords:** diabetes type ii, pulmonary tb (ptb), fungal ball, aspergillus, mtb (mycobacterium tuberculosis)

## Abstract

Pulmonary tuberculosis is rampant in some countries. The disease is an outcome of infection by *Mycobacterium tuberculosis* and is more common in immunocompromised individuals. Furthermore, mycetoma or a fungal ball can develop in cavitary lesions of tuberculosis. The present case is a rare presentation of pulmonary tuberculosis with concomitant *Aspergillus *fungal ball in a diabetic Indian male. A clinical examination with a strong laboratory and radiological workup helped establish the final diagnosis. The patient was initiated on anti-tubercular chemotherapy and advised lobectomy.

## Introduction

Tuberculosis is a disease with a direct impact on public health [1]. It results from an infection caused by *Mycobacterium tuberculosis *and can be either pulmonary or extrapulmonary [2]. The most common transmission mode of infection is by inhalation of the bacilli [2]. The disease has a proclivity for immunocompromised individuals [3]. The latest reports from the year 2021 mention an incidence and prevalence of the disease in India of 188 and 312 per one lakh (0.1 million) population, respectively [4].

Fungal infection of the lungs is relatively common in immunocompromised patients [5]. Oftentimes, this infection is clinically present as a fungal ball, which is also termed mycetoma [6]. Various species of fungi could result in the development of a fungal ball [6]. A fungal ball due to the *Aspergillus* species is a rare clinical presentation and is characterized by clumps of fungi in the tissue, usually in the lungs or sinuses, ending up with chronic inflammation and hemoptysis [2]. Primary aspergilloma is an infrequently reported condition, as this infection mostly occurs secondary to tuberculosis infection in preexisting cavities [7]. A coexistence of *Aspergillus* fungal balls in an active tuberculosis case is very rare [7].

The present case is a 47-year-old Indian male with a known history of diabetes who reported recurrent hemoptysis, cough with expectoration, and fever. In an endemic country for tuberculosis, he was diagnosed after a detailed laboratory and radiological workup and started on an anti-tubercular and antifungal treatment.

## Case presentation

A 47-year-old Indian male with a history of diabetes mellitus type II for two years reported chief complaints of recurrent hemoptysis, cough with expectoration, and fever for one month. He was well 30 days ago before he had a fever that rose in the evening and was alleviated after taking over-the-counter paracetamol. This fever initially occurred once every two to three days, but its frequency increased to daily for three weeks. He also had a cough with yellow-colored, non-foul-smelling expectoration for two weeks. Initially, the expectoration was not blood-tinged, but he had hemoptysis (about 50-100 mL of blood per episode) for one week. There was no remarkable loss of weight or hematemesis. Furthermore, he had no similar complaints in the past or a history of tuberculosis, even among his close contacts. There was no history of trauma, substance abuse, or any significant medical or surgical intervention in the past except for diabetes mellitus type II, for which he was taking oral hypoglycemic medications. In addition, he had worked at a cement factory for three years.

A general examination revealed an average-built male with normal vitals (oxygen saturation 99% in room air). Systemic examination was remarkable for decreased vesicular breath sounds on auscultation of the left hemithorax. Moreover, there was no lymphadenopathy, clubbing, pallor, jaundice, pretibial edema, or cyanosis. The fundus examination was normal.

Due to the endemicity of tuberculosis and classical features at presentation, along with a history of diabetes mellitus type II, a probable diagnosis of tuberculosis was made, and he was advised sputum smear microscopy for acid-fast bacilli (after prescribing a tablet of micronized purified flavonoid fraction 500 mg twice daily), a cartridge-based nucleic acid amplification test (CBNAAT) of the sputum, and a chest radiograph.

**Figure 1:**
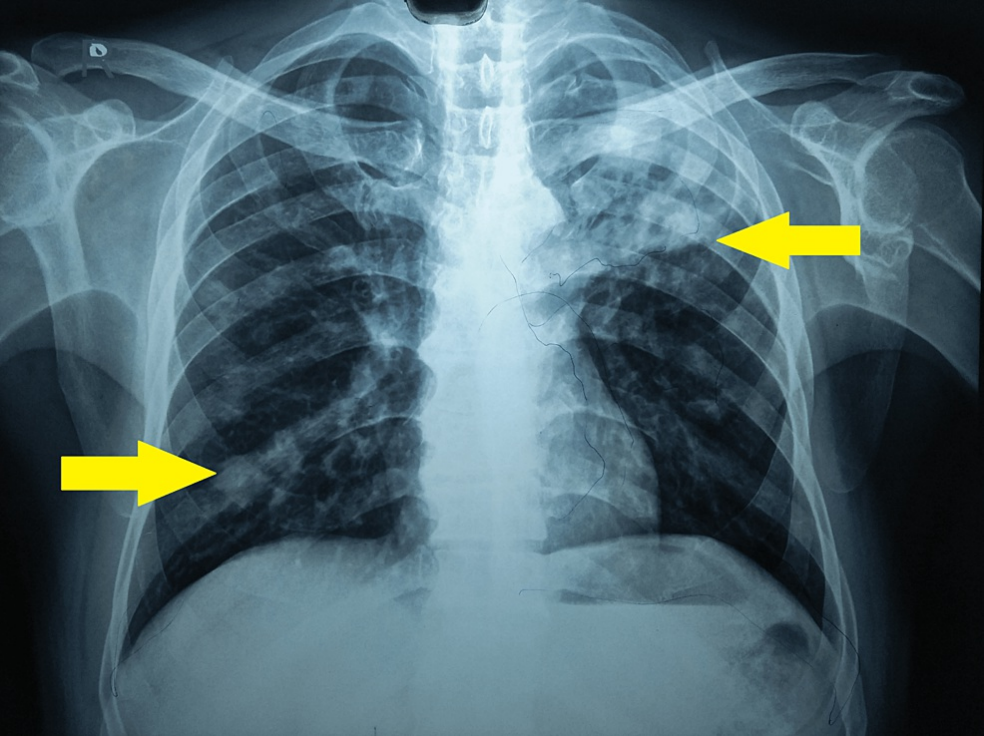
An antero-posterior chest radiograph showing bilateral involvement with a solid oval mass within a left upper lobe cavity and right lower lobe consolidation.

The results were suggestive of tuberculosis, with acid fast bacilli seen on the sputum smear microscopy and *Mycobacterium tuberculosis* detected on CBNAAT (low detection with no resistance to rifampicin). An antero-posterior chest radiograph revealed bilateral confluent and discrete patchy inhomogenous parenchymal infiltrates (left>right) with a solid oval mass within a left upper lobe cavity partly encircled by a radiolucent crescent (positive Monod’s sign) (Figure 1).

**Figure 2:**
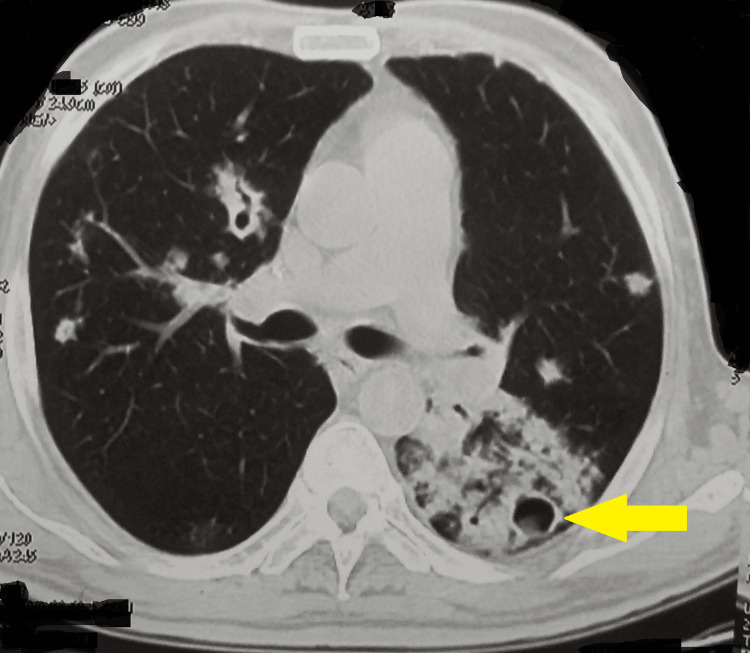
A non-contrast computed tomography of the chest showing a fungal ball with multiple varying-sized cavitary lesions.

The results of the line probe assay and sputum culture were suggestive of *Mycobacterium tuberculosis* with no resistance to first-line anti-tubercular drugs. A non-contrast computed tomography of the chest also showed the Monod sign in a left lung cavitary lesion with a gravity-dependent density suggestive of a fungal ball. It also showed multiple varying-sized cavitary lesions, with the largest one involving the left upper lobe and the apical segment of the left lower lobe showing associated collapse, consolidation, and soft infiltrates (Figure 2). Moreover, varying-sized mediatinal lymph nodes, namely, prevascular, pretracheal, precarinal, and subcarinal, were seen. A right hilar calcified node was noted (which could be due to his exposure to cement at work). A minimal overlying pleural thickening in the left upper chest posteriorly was noted.

A bronchoalveolar lavage was done, and the results show a positive galactomannan antigen test (0.54) (reference range: <0.5 = negative and ≥0.5 = positive) with the detection of *Aspergillus* species deoxyribonucleic acid on a polymerase chain reaction assay of bronchoalveolar lavage fluid. A fungus culture of bronchoalveolar lavage fluid showed the growth of *Aspergillus fumigatus*. Moreover, there was a high erythrocyte sedimentation rate of 45 mm in the first hour, a random blood sugar level of 180 mg/dl, and glycated hemoglobin of 7.5%. His tuberculin skin test result was positive, and his HIV I and II test results were non-reactive.

Ultimately, a diagnosis of pulmonary tuberculosis with* Aspergillus *fungal ball was made, and he was initiated on an anti-tubercular treatment per his weight (Table 1).

**Table 1:** Anti-tubercular treatment per his weight

Phase	Drug	Dose	Duration
Intensive phase	Rifampicin	10 mg/kg	56 days
	Pyrazinamide	25 mg/kg	56 days
	Ethambutol	15 mg/kg	56 days
	Isoniazid	5 mg/kg	56 days
Continuation phase	Rifampicin	10 mg/kg	112 days
	Ethambutol	15 mg/kg	112 days
	Isoniazid	5 mg/kg	112 days

His oral hypoglycemic medications were continued (a tablet of metformin hydrochloride 500 mg twice daily, a tablet of glimepiride 2 mg twice daily, and a tablet of pioglitazone 1 mg twice daily), and itraconazole (200 mg twice daily) antifungal therapy was initiated. After the therapeutic drug monitoring, his antifungal treatment was supposed to be substituted with other antifungal drugs (total of six months), with the possibility of modifying the anti-tubercular treatment based on drug-drug interactions. A tablet of pyridoxine (20 mg per day) was also added. He was also advised a left thoracotomy with an upper and lower lobectomy, but he was reluctant to undergo surgery. After initiation of the treatment, he started feeling better (no episodes of hemoptysis and reduction in cough and fever episodes), and even after repeated counseling, he did not give consent for surgery and was subsequently lost to follow-up.

## Discussion

*Mycobacterium tuberculosis *is a noteworthy contributor to morbidity and mortality, especially in Asia and Africa [1]. The disease has been known since ancient times and is usually seen with the weakening of the immune system [8]. The classical signs include fever, cough with expectoration, weight loss, and night sweats [1]. The situation becomes alarming when the patient presents with hemoptysis. Oftentimes, these hemoptysis episodes follow a cough episode [9].

Fungal infection of the lungs is usually seen in immunocompromised patients [5]. Most common *Aspergillus* species that causes invasive infections in humans is *Aspergillus fumigatus* [7,9]. The infection could be self-limiting, i.e., simple fungal colonization to a lethal variety that could be invasive [7]. Furthermore, *Aspergillus *can cause a wide variety of presentations, including simple colonization, allergic bronchopulmonary aspergillosis, invasive aspergillosis, and aspergilloma due to the growth of saprophytes [7].

The constituents of a fungal ball include both dead and alive mycelial elements, cellular elements, amorphous debris, and mucus [7]. The common clinical presentation is a cough, wheeze, shortness of breath, or loss of weight. The cough may be associated with a hemoptysis, which could prove lethal if left untreated, with fatalities around 2-14% [7]. It requires a detailed clinical examination with a high index of suspicion supported by strong radiographic evidence, along with serological or microbiologic evidence of *Aspergillus*, for the diagnosis [10,11]. Management is based on the type of presentation and varies from no treatment in asymptomatic cases to systemic or local administration of antifungal agents and/or surgical resection [7]. Other noteworthy management options may involve bronchial artery embolization or cavernostomy [7].

In the present case, the patient was initiated on itraconazole (due to financial implications), and therapeutic drug monitoring was planned. Voriconazole was not started initially, as it is a substrate and an inhibitor of CYP3A4, CYP2C9, CYP2B6, and CYP2C19 cytochromes, which limits its use due to a broad range of drug-drug interactions, including with anti-tubercular drugs, such as rifampicin. His antifungal treatment was supposed to be substituted per the drug-drug interactions. Options like a modified anti-tubercular treatment were also planned. However, this patient was lost to follow-up.

A case similar to this present case was published by Ortiz et al. in 2023 [2]. The present case shares a number of features with theirs, such as clinical features, a history of diabetes, radiograph findings, and the detection of *Aspergillus*. However, certain unique features that separate the two cases were the absence of a tuberculosis history, bronchial anthracosis, and the presence of an air-crescent sign in this case [2]. In addition, the present case was male, whereas their case involved a female patient, and the final outcome of their case was fatal, but this case was stable at the last follow-up.

Another case sharing similarities with the present case was published by Soewondo et al. [12]. The present case shares a number of similarities with theirs, such as the presence of cough, side of the lesion, findings on chest auscultation, chest radiograph findings suggestive of an air-crescent sign, and history of diabetes [12]. However, the absence of dyspnea in the present case and hemoptysis in theirs separates the two [12].

Adeyemo et al. reported successful management of nine among 11 cases with combined medical (anti-tubercular drugs) and surgical treatment [13].

A case of pulmonary tuberculosis with concomitant fungal balls in a diabetic Indian male is presented here. This case highlights the importance of a high degree of suspicion and a detailed diagnostic workup to establish a diagnosis. This case was also unique, as a fungal ball is commonly reported in secondary cavities of tuberculosis, lung cancer, cystic fibrosis, bullous emphysema, and pulmonary abscesses, and a simultaneous infection in a primary tuberculosis case is rare, even in endemic countries. Nevertheless, having a single case was a limitation here, but this case stresses the need for the availability of data from bigger centers in endemic countries to formulate new or modify existing guidelines for the management of both tuberculosis and aspergilloma.

## Conclusions

The present case is of a diabetic patient with pulmonary tuberculosis and concomitant aspergilloma. The co-occurrence of aspergilloma in active tuberculosis is rare, as fungal balls are usually present secondary to residual cavities in the lungs. Having diabetes is an immunocompromised condition, which increases the risk for both mycobacterial and fungal infections. Therefore, it is essential that clinicians have a high index of suspicion to diagnose and timely treat such cases because delays or missed treatments could prove lethal for the patient’s life.
